# Application of Extremely Randomised Trees for exploring influential factors on variant crash severity data

**DOI:** 10.1038/s41598-022-15693-7

**Published:** 2022-07-07

**Authors:** Farshid Afshar, Seyedehsan Seyedabrishami, Sara Moridpour

**Affiliations:** 1 Faculty of Civil & Environmental Engineering, Tarbiat Modares University, Tehran, Iran; 2 Civil and Infrastructure Engineering Discipline, RMIT University, Melbourne, Australia

**Keywords:** Risk factors, Engineering

## Abstract

Crash severity models play a crucial role in evaluating the influencing factors in the severity of traffic crashes. In this study, Extremely Randomised Tree (ERT) is used as a machine learning technique to analyse the severity of crashes. The crash data in the province of Khorasan Razavi, Iran, for a period of 5 years from 2013 to 2017, is used for crash severity model development. The dataset includes traffic-related variables, vehicle specifications, vehicle movement, land use characteristics, temporal characteristics, and environmental variables. In this paper, Feature Importance Analysis (FIA), Partial Dependence Plots (PDP), and Individual Conditional Expectation (ICE) plots are utilised to analyse and interpret the results. According to the results, the involvement of vulnerable road users such as motorcyclists and pedestrians alongside traffic-related variables are among the most significant variables in crash severity. Results show that the presence of motorcycles can increase the probability of injury crashes by around 30% and almost double the probability of fatal crashes. Analysing the interaction of PDPs shows that driving speeds above 60 km/h in residential areas raises the probability of injury crashes by about 10%. In addition, at speeds higher than 70 km/h, the presence of pedestrians approximately increases the probability of fatal crashes by 6%.

## Introduction

In recent years, injuries and casualties resulting from road crashes have been one of the leading death causes in Iran and stood as the third major death cause between 2005 and 2017. During an 8-year period from 2013 to 2020, more than 2 million people were seriously injured, and nearly 100,000 people died in traffic crashes in Iran^1^. The large number of crash fatalities and severe injuries also impose a significant economic burden. According to the^2^, in 2016, the cost of fatalities and serious injuries in Iran was around $28.5 billion accounts for 6.8% of the country’s Gross Domestic Product (GDP).

Khorasan-Razavi, in the northeast of the country, is the second most populated province in Iran and is chosen by many people as a leisure trip destination every year. Khorasan-Razavi has one of the highest crash casualties on rural roads across provinces. Between 2013 and 2020, 7040 fatalities and 165,339 severe injuries were observed on rural roads in this province, which both were the second-highest in the country. These statistics suggest a need for an in-depth understanding of the key factors contributing to crash severity in rural areas. However, there are few studies in which real-time traffic data is used to analyse the severity of crashes in rural areas in developing countries.

According to the literature, two approaches are available to identify the influencing factors on crash severity, including statistical models and data-driven methods. Statistical models are the primary method in crash frequency and severity analysis. These models have specific assumptions about model configuration and the used datasets. For instance, there is a linear additive assumption regarding the relationship between independent and dependent variables, which may not be completely accurate in some cases. This functional form can have a negative impact on results due to outliers and missing values^3–5^. In addition, due to the temporal instability of parameters in traditional statistical models, the capabilities of these models in predicting crash occurrence and crash severity are limited. To overcome this limitation, the traditional statistical models have been extended to endogeneity and heterogeneity models. However, dimensionality challenges have been observed for large datasets when endogeneity and heterogeneity models are used^6,7^. Data-driven methods include a variety of techniques, among them Machine Learning (ML) techniques, which have progressed drastically over the past years. ML methods have few or no pre-assumed relationship between covariates, and they can better handle outliers as well as the missing and noisy data^8,9^. There is a growing body of research on the analysis of road crash severity using ML techniques. In these studies, ML techniques are mainly used for the prediction of crash severity, including artificial neural networks^10–14^, tree-based models^4,5,8,15–25^, Support Vector Machines (SVM), K-Nearest Neighbour (KNN), clustering methods, Naïve Bayes (NB), and hybrid models^3,20,26–30^. Heuristic and metaheuristic methods are also used in a few studies, and their results have been compared with machine learning-based models^31,32^.

Crash severity data are usually disproportionally distributed. The response variable is highly variated and imbalanced towards Property Damage Only (PDO) and less severe crashes. Traditional classification machine learning algorithms have a bias toward majority classes. However, due to the importance and the higher costs of severe and fatal crashes, there will be a significant loss in case of misclassification of severe and fatal crashes^33^. To address this problem, there are two solutions, namely data-level and algorithm-level solutions. At data-level solutions, usually, different sampling methods are used to create a balance between different classes of data. At algorithm-level methods, an ensemble methodology is used to improve the performance of a single classifier. In this study, the latter has recruited to deal with imbalanced and high variance of the used crash data.

The traffic profile data used in traditional crash severity models as independent variables are aggregate and are mainly based on static traffic data. These types of data are unable to explain variations in traffic profiles prior to the crash occurrence^34^. By emerging high-resolution traffic data, it is possible to capture detailed individual drivers’ behavioural information and other influencers in traffic crashes^35^. By analysing microscopic driving behaviour through high-resolution data, it is possible to investigate movement variations before the crash^36^.

To enhance model robustness and improve the overall performance of the system, different ensemble methods have been recruited in the context of supervised learning. Dietterich^37^ introduced ensemble methods as learning algorithms that develop a set of classifiers/regressors to classify/regress new data points by taking a vote of their predictions. In ensemble learning, a set of base learners are developed, and then, by combining them, a composite predictor is created^38,39^. Ensemble learning is also widely used in crash severity analysis. Geurts, and Louppe^40^ developed a vote sequential minimal optimisation algorithm (an improved training algorithm based on SVMs) with bagging decision trees to analyse fatality occurrence in Lebanese road crashes, which outperformed sequential minimal optimisation, Naïve Bayes, logistic regression, artificial neural networks, Random Forest (RF), and bagging decision trees. Effati et al.^20^ compared four models to predict motor vehicle crashes and their severity on two-lane, two-way roads, including Classification And Regression Tree (CART), Fuzzy Classification And Regression Tree (FCART), bagged-FCART, and SVM. They found that bagged-FCART provides higher overall accuracy and Kappa in comparison to the other three models. RF technique is one of the widely used ensemble learning methods in crash severity analysis. Mafi et al.^4^ and Zhang et al.^8^ used RF models in comparison to ordered probit, multinomial logit, K-nearest neighbour, decision tree, and SVM to predict the crash severities in Florida, U.S. Tang et al.^9^ proposed a newly introduced two-layer stacking framework to analyse crash severity with 3-year crash data from 2004 to 2006 of Florida, U.S. In the first layer, three classifiers, including random forests, AdaBoost with decision tree base estimator, and Gradient Boosting Decision Tree (GBDT), were trained and then based on the outputs of the first layer, logistic regression was fitted as meta classifier in the second layer. Results showed that the stacking model had more accurate predictions compared to SVM, multi-layer perceptron and RF. In random forests, by randomly associating variables to each tree, the variance of the results will be reduced. However, in tree-based models, there are significant error rates originating from cut-point (the splitting threshold for each tree at each node) variance^41^. In random forests, the cut points are selected to optimise the quality of the split (i.e. Gini impurity or entropy), and therefore it cannot capture the cut-point variance. To overcome this limitation, Extremely Randomised Trees (ERTs) have been introduced by^41^. In contrast to RFs that use bootstrap sampling (i.e. random sampling with replacement), ERT uses the whole sample. Multiple studies in different fields like computer science, electrical engineering, chemical engineering, and energy systems have shown that ERT often can be considered a competitive alternative to RFs in terms of accuracy and other goodness of fit measures^42–46^. Crash data inherently contains different sources of variance. Variations in vehicle attributes, roadway characteristics, and environmental conditions affect both the likelihood and resulting injury severity of crashes. In addition, variance in drivers’ physiologies and responses to the aforementioned sources can have an impact on crash severity^47^. With such highly variant data, ERT’s ability to capture variances in data may result in better evaluation and prediction of the severity of crashes.

This paper contributes to the safety literature by providing an ERT for analysing inter-urban traffic crashes in Iran between 2013 and 2017. The crash data of rural roads in Khorasan Razavi, Iran, is used in this research. No previous research in the literature used ERT for analysing crash severity in rural areas. In this research, five types of variables, including traffic variables (e.g. average speed, traffic flow), vehicle types (e.g. motorcycle at fault), vehicle movement (e.g. moving forward, left turn), land use specifications (e.g. residential, agricultural), temporal characteristics (e.g. month, season), and environmental variables (e.g. road lighting, road and shoulder pavement condition, road surface condition) are used to develop the model.

The rest of this paper is structured as follows. The case study, including the data description, is presented in the following section. It is followed by explaining the specifications of ML classifiers and presenting the model. After that, the results are presented, and the findings are discussed in “Results and discussions” section. The final section presents concluding remarks and highlights directions of future research.

## Dataset

In this study, the crash dataset of rural roads in Khorasan Razavi, Iran, is used. The data is obtained from the Road and Maintenance Transportation Organisation in Iran for a period of 5 years, from 2013 to 2017. The major part of the crash data is extracted from police reports. The police reports contain information about injury severity, road geometry (e.g. shoulder type), weather condition, environmental condition (e.g. road lighting), crash cause, and vehicles and individuals involved in a crash. Real-time traffic data collected by inductive loop detectors are also used in this research. The mean and standard deviation of loop detectors’ speed, headway, and hourly traffic volumes are used in this research to represent real-time traffic situations before the occurrence of a crash. In this research, the crash severity is categorised into three ordered levels, including PDO, injury, and fatal. These levels are shown in Table 1.

**Table 1:** Injury severity categories in the crash dataset.

Severity level		Train	Test	Total
Level 1	PDO-no injury	424 (37%)	104 (36%)	528 (37%)
Level 2	Injury	674 (59%)	166 (58%)	840 (59%)
Level 3	Fatal injury	43 (4%)	16 (6%)	59 (4%)
Overall		1141	286	1427

Level 1 shows the property damage only crashes, accounting for 37% of observations. Level 2 includes all kinds of injuries that occur at the crash scene. Due to the wide variation of injury (from anticipating injury to possible injury), this level is the largest component of the data (59%). Level 3 belongs to fatal crashes, which has the lowest percentage (around 4%). 31 variables, including 28 binary variables and 3 numeric variables, are selected for modelling. A detailed description of these variables is shown in Table 2.

**Table 2:** Description of the explanatory variables.

Category	Variable	Type	Description	Train	Test
Vehicle	Moving forward	Binary	Crash occurred while moving forward		
			Zero	508 (45%)	120 (42%)
			One	633 (55%)	166 (58%)
	Motorcycle at fault	Binary	Motorcycle hit another vehicle		
			Zero	1034 (91%)	260 (91%)
			One	107 (9%)	26 (9%)
	Car involved in crash	Binary	At least one passenger car involved in crash		
			Zero	817 (72%)	212 (74%)
			One	324 (28%)	74 (26%)
	Hit fixed objects	Binary	Vehicle hit fixed objects		
			Zero	1044 (91%)	265 (93%)
			One	97 (9%)	21 (7%)
	Left turn	Binary	Crash occurred when one vehicle turning left		
			Zero	1079 (95%)	271 (95%)
			One	62 (5%)	15 (5%)
	Motorcycle innocent	Binary	Motorcycle was hit by another vehicle		
			Zero	1022 (90%)	254 (89%)
			One	119 (10%)	32 (11%)
	Truck at fault	Binary	Truck was found at fault in crash		
			Zero	1037 (91%)	260 (91%)
			One	104 (9%)	26 (9%)
	Truck innocent	Binary	Truck was not found at fault in crash		
			Zero	1082 (95%)	277 (97%)
			One	59 (5%)	9 (3%)
	Pedestrian involved	Binary	Pedestrian was hit by vehicle		
			Zero	1057 (93%)	259 (91%)
			One	84 (7%)	27 (9%)
Land use	Residential area	Binary	Crash occurred in residential area		
			Zero	869 (76%)	219 (77%)
			One	272 (24%)	67 (23%)
	Agricultural area	Binary	Crash occurred in agricultural area		
			Zero	1055 (92%)	268 (94%)
			One	86 (8%)	18 (6%)
Crash cause	Fail to keep longitudinal distance	Binary	Fail to keep longitudinal distance		
			Zero	1077 (94%)	272 (95%)
			One	64 (6%)	14 (5%)
	Failing to yield the right of way	Binary	Driver failed to yield the right of way		
			Zero	1019 (89%)	260 (91%)
			One	122 (11%)	26 (9%)
Temporal	February	Binary	Crash occurred in February		
			Zero	1083 (95%)	262 (92%)
			One	58 (5%)	24 (8%)
	Winter	Binary	Crash occurred in winter		
			Zero	854 (75%)	199 (70%)
			One	287 (25%)	87 (30%)
Environment	No road marking	Binary	No marking condition was in the crash scene		
			Zero	937 (82%)	224 (78%)
			One	204 (18%)	62 (22%)
	Dry road surface	Binary	Dry road surface		
			Zero	323 (28%)	90 (31%)
			One	818 (72%)	196 (69%)
	Unpaved shoulder	Binary	Road had unpaved shoulder at crash scene		
			Zero	890 (78%)	203 (71%)
			One	251 (22%)	83 (29%)
	Clean weather (no cloud)	Binary	No cloud was in the sky		
			Zero	228 (20%)	73 (26%)
			One	913 (80%)	213 (74%)
	Road lighting deficiency	Binary	Lighting system was not fully functional		
			Zero	1133 (99%)	283 (99%)
			One	8 (1%)	3 (1%)
	Collision in the median strip	Binary	Collision occurred in the median strip		
			Zero	1052 (92%)	266 (93%)
			One	89 (8%)	20 (7%)
	Broken line	Binary	Broken marking line		
			Zero	402 (35%)	123 (43%)
			One	739 (65%)	163 (57%)
	Direct-downhill road	Binary	Crash occurred in a direct-downhill road		
			Zero	1106 (97%)	278 (97%)
			One	35 (3%)	8 (3%)
	Night without enough lighting	Binary	Crash occurred at night without enough lighting		
			Zero	892 (78%)	223 (78%)
			One	249 (22%)	63 (22%)
	Snowy and frozen surface	Binary	Snowy and frozen road surface		
			Zero	1119 (98%)	277 (97%)
			One	22 (2%)	9 (3%)
	Paved shoulder	Binary	The road had paved shoulder at crash scene		
			Zero	581 (51%)	165 (58%)
			One	560 (49%)	121 (42%)
Traffic	Flow rate (vehicle/min)	Continues	Traffic flow rate one hour before crash		
			Mean	8.07	8.1
			Standard deviation	8.34	8.44
	Headway (sec)	Continues	Headway one hour before crash		
			Mean	37.12	42.42
			Standard deviation	183.66	237.63
	Average speed (km/h)	Continues	Average speed one hour before crash		
			Mean	80.05	79.53
			Standard deviation	15.28	14.82

## Model development and interpretation

In this study, ERTs investigate the relationship between crash severity and the associated influencing factors. Similar to the RF model, the ERT is an ensemble technique in which a large number of decision trees are developed, and the most accurate tree is selected by the majority vote. In the following subsections, the ERT technique is explained.

### Extremely Randomised Trees (ERTs)

The ERT is a tree-based ensemble technique. In this technique, a set of classification trees are developed. In this study, Classification And Regression Trees (CARTs) are used as the base decision tree algorithm. In comparison to other decision tree algorithms like Iterative Dichotomiser 3 (ID3) and C4.5, CART can deal with both numerical and categorical variables, identify the most significant variables and eliminate insignificant ones, and handle outliers^48^. In this study, there are both numerical and categorical variables, and due to a large number of input variables, some of the insignificant variables should be discarded. In ERT, at each node, a random subset of K variables is selected from all input variables to decide on the split. Every tree is developed from the complete learning sample. Using the complete sample instead of bootstrap copying and random selection of cut-points are two key distinctive properties of ERT. The ERT technique has a number of parameters that should be tuned, among which three of them are more important. Those three parameters include: (1) the number of attributes that determine the strength of the future selection process (denoted by K), (2) the minimum sample size for splitting a node that controls the strength of averaging output noise (shown by n_min_), and (3) the number of trees (represented by M) that decides variance reduction of the ensemble model^41,49,50^. For prediction, ERT similar to other ensemble methods, uses the combination of prediction results from each base model to predict the results for unseen data. In case of classification problems each base model contributes a vote for a category, and the category with the most votes wins for a given record in the list (majority rule)^51,52^.

### Tuning of parameters

The cross-validation method is used to find the optimum values for the above mentioned three parameters. In addition to those 3 parameters, Minimum impurity Decrease (MiDe) is tuned. MiDe controls the depth of trees while fitting decision trees. The most accurate performing combination of these parameters is found through a grid search between all possible combinations. 5 values of M, 10 values of n_min_, two values of K ($\sqrt{{\varvec{p}}}$ recommended by^42^, ${\mathrm{log}}_{2}{\varvec{p}}$) and 10 values of MiDe result in 1000 possible combinations of parameters. These combinations are examined in Python 3.6 via Scikit learn, Pandas, Numpy, and Matplotlib libraries.

### Model assessment

In this study, the dataset is divided into train and test data to develop the ERTs. 80% of the dataset is randomly selected for training, and the remaining dataset is selected for testing. To evaluate the accuracy of results, four well-known measures extracted from the confusion matrix are used, including (1) Accuracy, (2) Precision, (3) Recall, and (4) F-measure. These measures are presented as follows.

1 $$Accuracy=\frac{True \; Positive+True \; Negative}{True\; Positive+True \;Negative+False \;Positive+False \;Negative}$$

2 $$Precision =\frac{True \;Positive}{True \;Positive+False\; Positive}$$

3 $$Recall=\frac{True \;Positive}{True \;Positive+False \;negative}$$

4 $$F{\text{-}}measure=\frac{2\times Precision\times Recall}{Precision+Recall}$$

Accuracy (Eq. 1) is the most common measure for classification problems^53,54^. The quality of the model is evaluated by accuracy through the proportion of all correct classifications over all observations. Accuracy is easily computable, understandable, and applicable for multi-class problems. However, this metric has some limitations. Due to the less distinctive value, it shows poor discriminating power in selecting an optimal classifier between a subset of models. In addition, it is inclined to majority class instances. Precision (Eq. 2) is the fraction of the valid classification of one class among predicted instances of that class. Recall (Eq. 3) is the ratio of retrieved and relevant observations to all relevant observations. Precision gives information on how well the model performs with regard to the False Positives. Recall, similar to precision, provides information about the performance with regards to the False Negatives. F-measure (Eq. 4), which is the harmonic mean, combines Precision and Recall. In comparison to accuracy, F-measure has a more distinctive power.

### Model-agnostic methods for model interpretation

#### Feature importance

In tree-based models, the decrease in node impurity is weighted according to the ratio of the number of samples that reach a node to the total number of samples. This ratio is considered as feature importance. In a single decision tree model, the importance of a node is computed as follows.

5 $${node\_imp}_{i}={w}_{i}{c}_{i}-{w}_{l\left(i\right)}{c}_{l\left(i\right)}-{w}_{r\left(i\right)}{c}_{r\left(i\right)}$$

where: node_imp_i_: importance of node *i*, *w*_*i*_: weighted number of samples reaching node *i*, *c*_*i*_: the impurity value of node *i*, *l(i)*: child node from left split on node *i*, *r(i)*: child node from right split on node *i*.

Then importance of a feature in a decision tree is calculated using Eq. (6).

6 $${feat\_imp}_{j}=\frac{\sum_{i:node \; i \;splits \;on \;feature \; j}{node\_imp}_{i}}{{\sum }_{k\in I}{node\_imp}_{k}}$$

where: *feat_imp*_*j*_: importance of feature *j*, *node_imp*_*i*_: importance of node *i*, *I*: set of nodes.

Normalised values of feature importance can be derived using the sum of all feature importance values.

#### Partial dependence plots (PDPs)

Friedman^55^ introduced PDPs in the context of gradient boosting machines. PDPs depict the behaviour of the dependent variable as a function of the selected independent variable(s). This is similar to the interpretation of coefficients from multiple linear regression. However, using PDPs make it possible to capture more complex relationships instead of a fixed coefficient^56^. To visualise the dependence of response variable and input variables and due to difficulties at higher dimensional arguments, partial dependence of response variable is defined as follows.

7 $${f}_{S}({X}_{S})={E}_{{X}_{S}}f({X}_{S},{X}_{C})$$

where: ${f}_{S}$: marginal average of f over the distribution of feature ${X}_{C}$; ${X}_{S}$: Subvector of variables of interest; ${X}_{C}$: Subvector of variables in which *C* is the complement set for *S*, $S\cup C=\{\mathrm{1,2},\dots p\}$ and p represents number of input variables.

Usually, ${X}_{S}$ contains only one or two features and remaining appear in ${X}_{C}$. By averaging over all observations in train data partial dependence function can be estimated as:

8 $${\overline{f} }_{S}({X}_{S})=\frac{1}{N}\sum_{i=1}^{N}f({X}_{S},{x}_{iC})$$

where, {${x}_{1C},{x}_{2C},\dots , {x}_{NC}\}$ are the values of ${X}_{C}$ in train data^38,57^.

#### Individual conditional expectation

To disaggregate PDPs into individual levels, Goldstein et al.^58^ introduced Individual Conditional Expectation (ICE) plots which display the estimated functional relationship for each observation. This individual-level estimation can capture the potential individual heterogeneity. Instead of drawing a global plot for all observations (in PDPs), ICE presents the visual relationship between the dependent variable and the variable of interest, which results in multiple lines per variable. The values for each line are computed by fixing all variables and drawing new values for the variable of interest from a grid and making predictions based on these new values^57^. The conditional expectation curve is defined as follows.

9 $${\overline{f} }_{S}^{i}({X}_{S})=f({X}_{S},{x}_{iC})$$

Since every ICE plot has its own intercept, it is difficult to distinguish heterogeneity in the model. To solve this problem, Goldstein et al.^58^ proposed centred-ICE plots. In centred-ICE plots, a location ${x}^{*}$ in the range of ${X}_{S}$ is chosen and all predicted lines pinched to that point. It is shown that extremum values for ${x}^{*}$ gives the most interpretable plots. For each ${\overline{f} }^{i}$, the corresponding centred-ICE plots curve is given by Eq. (10).

10 $${\overline{f} }_{centered}^{i}={\overline{f} }^{i}-1({x}^{*},{X}_{iC})$$

where: $\overline{f }$: fitted model, $1$: vector of 1’s.

If ${x}^{*}$ id the minimum values of ${X}_{S}$, it is enough for curve to originate at 0.

## Results and discussions

### ERT model

**Figure 1:**
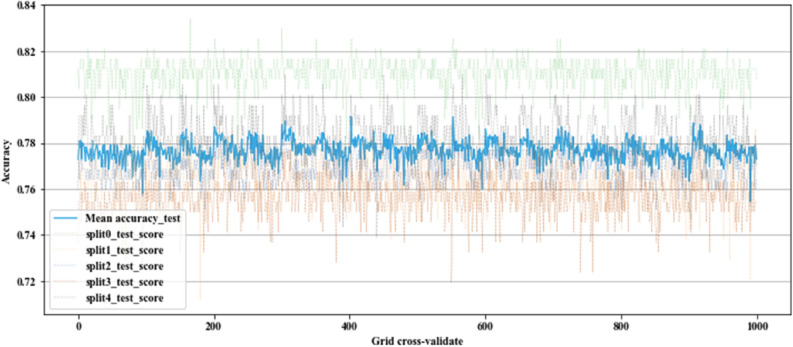
Accuracy score on different combination of hyper-parameters [plotted by Python Matplotlib v. 3.3.4 https://matplotlib.org].

In the ERT model development, 80% of the dataset is randomly selected as the train data to train the model, and the remaining 20% of the data is used as test data to examine the performance of the developed model. A cross-validation procedure is used to find the optimal parameters. In this procedure, the training dataset is divided into 5 smaller folds, and for every fold, a model is trained using fourfolds as training data and then the trained model is validated on the remaining fold. The average performance of this fivefold cross-validation is reported as model performance on the trained model. For each previously mentioned combination of parameters, a cross-validation procedure is executed. In Fig. 1, the results of the accuracy score for each combination of hyper-parameters are depicted. It can be seen that the model accuracy varies fold to fold and swings slightly between 0.72 and 0.84 (Fig. 1). The best set of parameters is achieved via {M: 141, MiDe: 0.0, K: $\sqrt{{\varvec{p}}}$, nmin: 11}.

To further evaluate the performance of the ERT model, a RF model is also developed, and the results from the RF model are compared with the results from the ERT model. Using a similar approach as the ERT model, fivefold cross-validation via the same set of hyperparameters has been recruited to calibrate the RF model. The comparison of the two models shows that the ERT model can outperform the other model, and the ERT model can be considered as a reliable competitor for RF models in crash severity analytics.

In Table 3, the final performance of the most accurate model is reported for test and train. By recruiting this set of parameters, the accuracy of 0.850 for the train and the accuracy of 0.814 for the test sets is achieved. Other measures also showed that there is little difference between the performance of the model on the train and test sets.

**Table 3:** Performance of selected models.

	Train			
	AdaBoost	XGBoost	Random forest	ERT
Confusion matrix	$$\begin{array}{cc}\begin{array}{c}PDO\\ Injury\\ Fatal\end{array}& \left[\begin{array}{ccc}291& 133& 0\\ 98& 575& 0\\ 8& 35& 0\end{array}\right]\end{array}$$	$$\begin{array}{cc}\begin{array}{c}PDO\\ Injury\\ Fatal\end{array}& \left[\begin{array}{ccc}282& 142& 0\\ 70& 604& 0\\ 6& 37& 0\end{array}\right]\end{array}$$	$$\begin{array}{cc}\begin{array}{c}PDO\\ Injury\\ Fatal\end{array}& \left[\begin{array}{ccc}310& 114& 0\\ 38& 636& 0\\ 5& 35& 3\end{array}\right]\end{array}$$	$$\begin{array}{cc}\begin{array}{c}PDO\\ Injury\\ Fatal\end{array}& \left[\begin{array}{ccc}326 & 98 & 0\\ 36& 638& 0\\ 5& 30& 8\end{array}\right]\end{array}$$
Accuracy	0.756	0.776	0.831	0.850
Precision	0.729	0.748	0.842	0.858
Recall	0.756	0.776	0.831	0.850
F-measure	0.743	0.758	0.816	0.840

	Test			
	AdaBoost	XGBoost	Random forest	ERT
Confusion matrix	$$\begin{array}{cc}\begin{array}{c}PDO\\ Injury\\ Fatal\end{array}& \left[\begin{array}{ccc}76& 28& 0\\ 14& 149& 3\\ 3& 13& 0\end{array}\right]\end{array}$$	$$\begin{array}{cc}\begin{array}{c}PDO\\ Injury\\ Fatal\end{array}& \left[\begin{array}{ccc}73& 31& 0\\ 9& 157& 0\\ 1& 15& 0\end{array}\right]\end{array}$$	$$\begin{array}{cc}\begin{array}{c}PDO\\ Injury\\ Fatal\end{array}& \left[\begin{array}{ccc}74 & 30& 0\\ 8& 158& 0\\ 1& 15& 0\end{array}\right]\end{array}$$	$$\begin{array}{cc}\begin{array}{c}PDO\\ Injury\\ Fatal\end{array}& \left[\begin{array}{ccc}76 & 28& 0\\ 8& 157& 1\\ 1& 15& 0\end{array}\right]\end{array}$$
Accuracy	0.787	0.805	0.811	0.815
Precision	0.752	0.769	0.775	0.781
Recall	0.787	0.804	0.811	0.815
F-measure	0.766	0.778	0.784	0.790

The comparison of confusion matrixes shows the ERT model generally outperforms the RF, XGBoost, and AdaBoost models. Focusing on less-frequent crash severity classes (e.g. fatal and PDO classes in this dataset), the ERT model slightly gained more accurate results than the other counterparts. Results from the ERT model show that this model, in comparison with the other models, is more inclined to the fatal class with the lowest frequency. The goodness of fit measures calculated in Table 3 indicates that the ERT model resulted in more accurate performance than its competitors even though the difference in measure values is low. These results show that the ERT model can be considered a competitive alternative for the RF, XGBoost, and AdaBoost models in classifying crash severity data.

**Figure 2:**
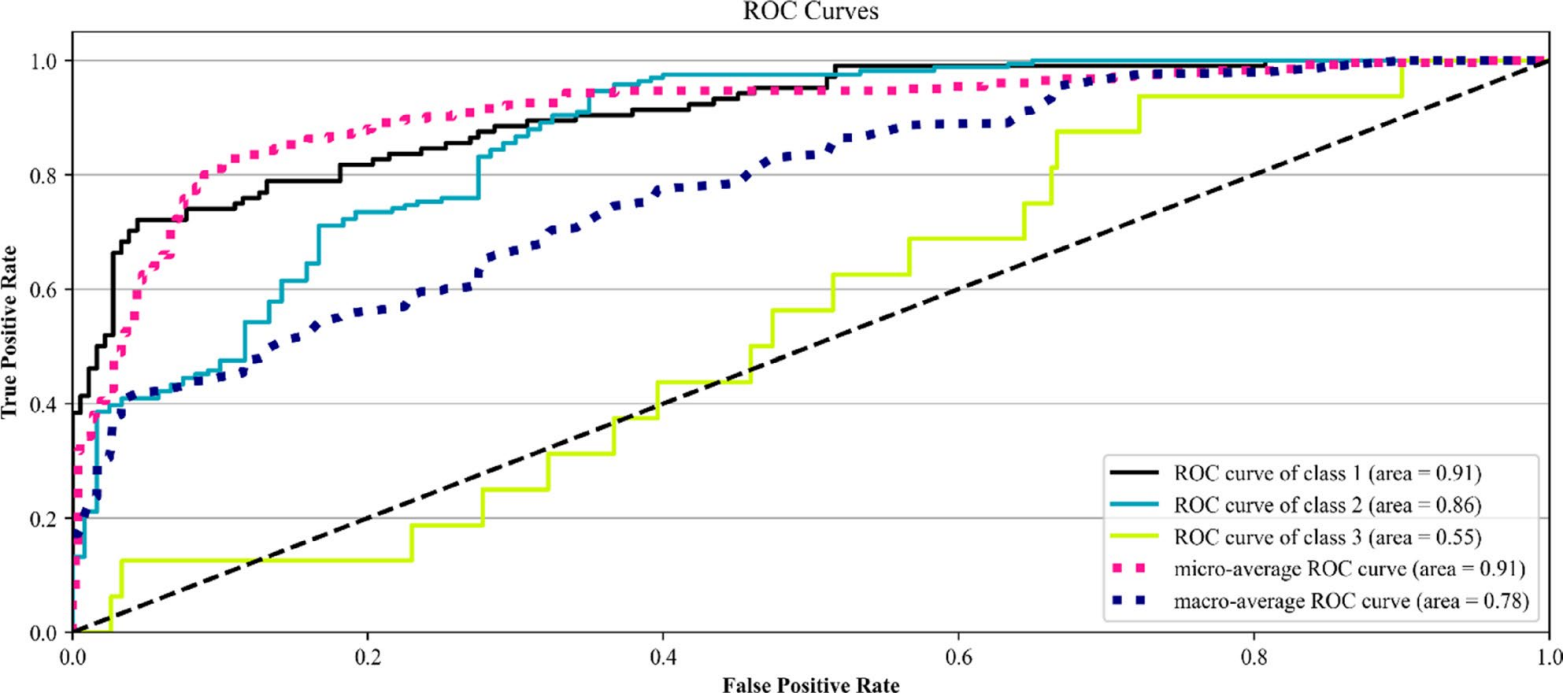
ROC curves for the ERT model [plotted by Python Matplotlib v. 3.3.4 https://matplotlib.org].

Figure 2 shows Receiver Operating Characteristics (ROC) curves for each class, as well as the micro-average and macro-average curves. In Fig. 2, the nearest curve to random guess belongs to class 3 (fatal injury). However, two other classes (class 1 for PDO and class 2 for injury) gained higher true positive rates.

### Feature importance analysis

**Figure 3:**
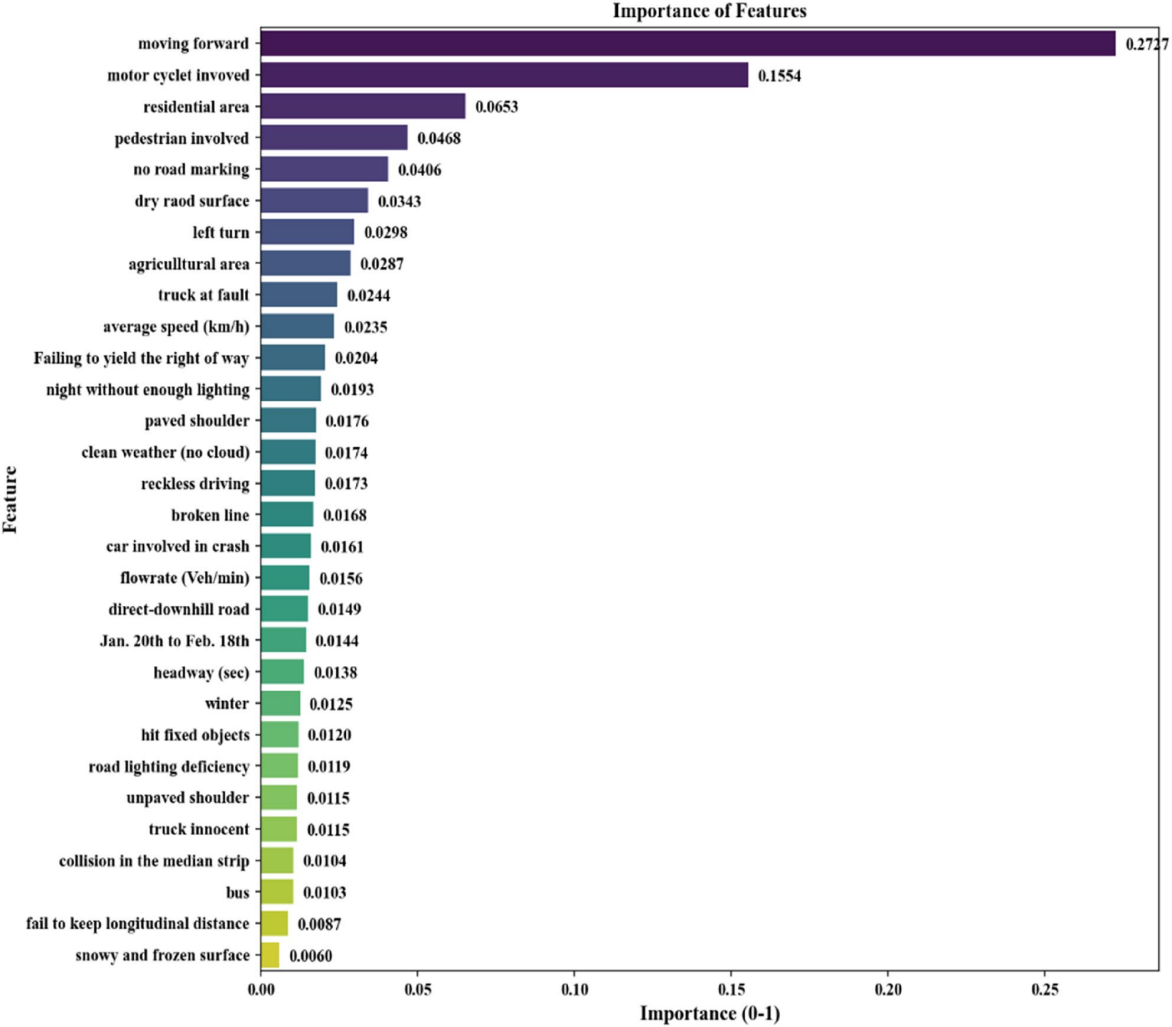
Importance of variables used in model [plotted by Python Matplotlib v. 3.3.4 https://matplotlib.org].

The importance of variables used in model development is calculated using Eq. (6) and is shown in Fig. 3. According to the results, “moving forward” is the most important variable in the model. Moving forward as a variable indicating the direction affects collision type and consequently severity of the crash. This is consistent with the results from previous studies^59^. Vulnerable road users like motorcyclists and pedestrians are among the important variables in the model. Encountering vulnerable road users, especially in rural areas, can lead to more severe crashes. Similar outcomes resulted in previous studies^60,61^. Land use of crash location is another influencing variable on crash severity. Residential and agricultural areas are two main land use types in rural areas, which are also ranked 3rd and 8th in the list of important variables. In rural roads of Iran, the probability of facing distracted pedestrians and bicyclists and slowly moving agricultural machinery in such areas are higher. This is consistent with two other studies by^16^ and^62^, in which Iranian and Malaysian crash data sets were recruited to develop decision trees and generalised ordered probit models to predict the crash severities.

One of the interesting results that can be derived from Fig. 3 is the importance of traffic-related variables. The average speed of traffic is found to be more important than 21 other variables. Limited studies have used traffic-related variables in crash severity analysis. In crash severity analysis conducted by^28^ and^63^, traffic-related variables obtained higher importance.

Environmental variables are also among the influencing variables in crash severity in this research. For instance, no road marking and dry road surface are among the most important variables influencing crash severity in rural areas. However, the snowy and frozen surface is the least important variable in crash severity.

It can be seen that variables like road light deficiency and snowy surface with a very limited share in the dataset have lower values of feature importance, but these variables are very effective in identifying fatal crash categories and removing them from the data set significantly affects performance metrics of model. For instance, by removing road light deficiency and snowy surface, accuracy and precision in the train dataset would drop by 6 and 10%, respectively.

### Partial dependence plot (PDP)

**Figure 4:**
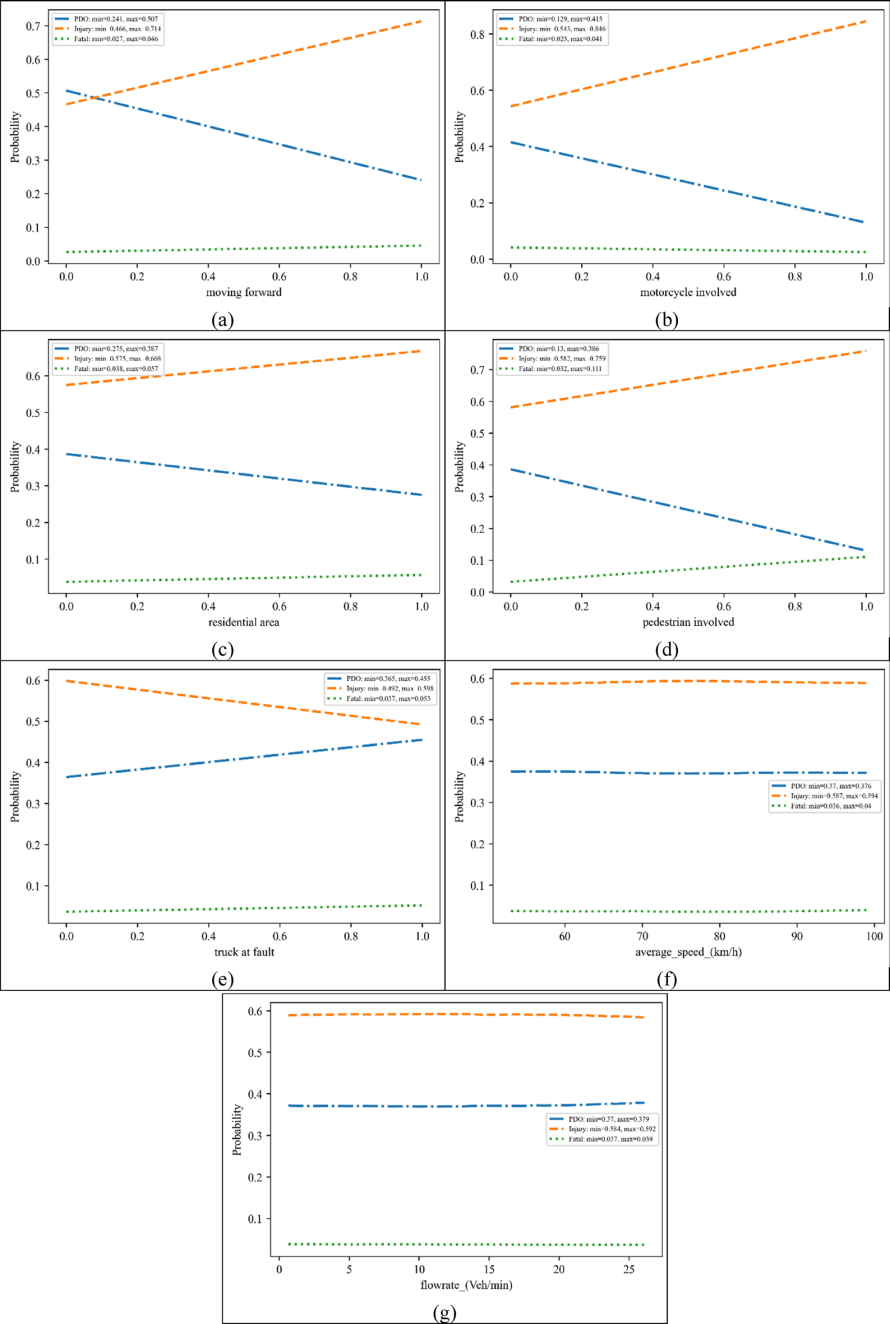
PDPs for the most influencing variables on crash severity [plotted by Python Matplotlib v. 3.3.4 https://matplotlib.org].

PDPs for the top important variables are depicted in Fig. 4. From Fig. 4a, it can be seen that “moving forward” increases the probability of an injury crash. The proportion of fatal crashes is significantly low, but “moving forward” increases the probability of fatal crashes from 2.5 to 4.7%. Figure 4b shows that if a motorcycle gets involved in a crash, the probability of the occurrence of injury crashes is increased. Different safety strategies are applied in urban areas in Iran to improve the safety of motorcyclists. However, strategies need to be implemented to enhance the safety of motorcyclists in rural areas. In Fig. 4c, it can be seen that the existence of a residential section can raise the probability of a more severe crash. As mentioned in the previous section, in residential sections of rural areas, the chance of being faced with a distracted pedestrian or bicyclist is higher. It can be seen from Fig. 4d that if a pedestrian is hit by a vehicle, the probability of fatal crashes significantly increases. It emphasises that a series of appropriate countermeasures should be taken to address non-motorised safety on rural roads. A controversial result that can be concluded from Fig. 4e is the growth of PDO probability and reduction of the probability of injury crashes by the involvement of the “trucks at fault” variable. When a truck involves in a crash, there will be two possible collisions, including (1) a very severe collision that would end up in a fatal crash; (2) a non-significant collision in which no one is hurt. These trends are shown in Fig. 4e. With regard to traffic-related variables, it is noticeable that these variables have a slight impact on the probability of each crash category. The impact of average speed (Fig. 4f) on different categories of crashes is less than 1%. In traffic flows higher than 22 vehicles per minute (1,320 veh/h) (Fig. 4g), the probability of injury crashes reduces by almost 1%.

### Individual conditional expectation (ICE) plots

**Figure 5:**
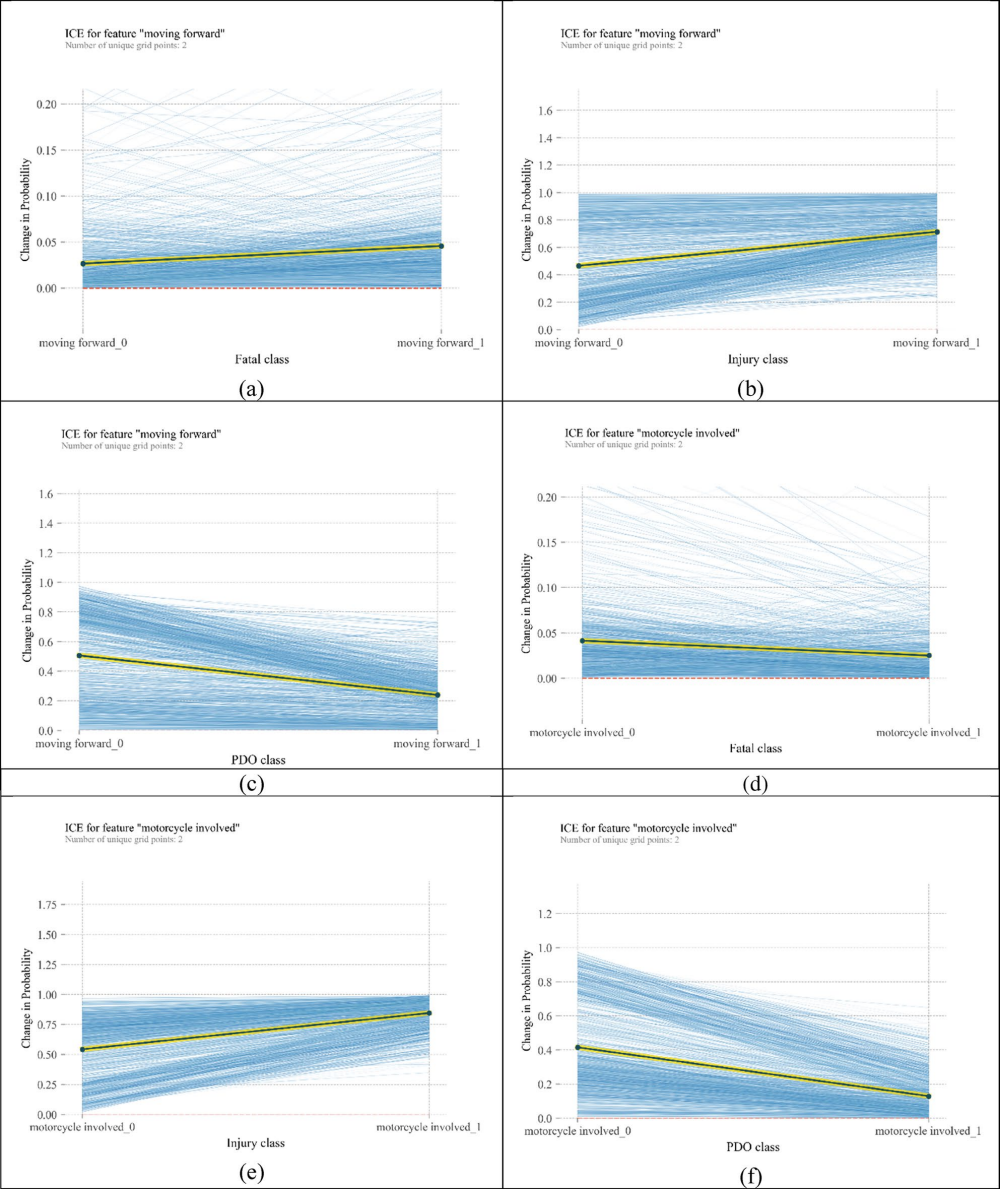

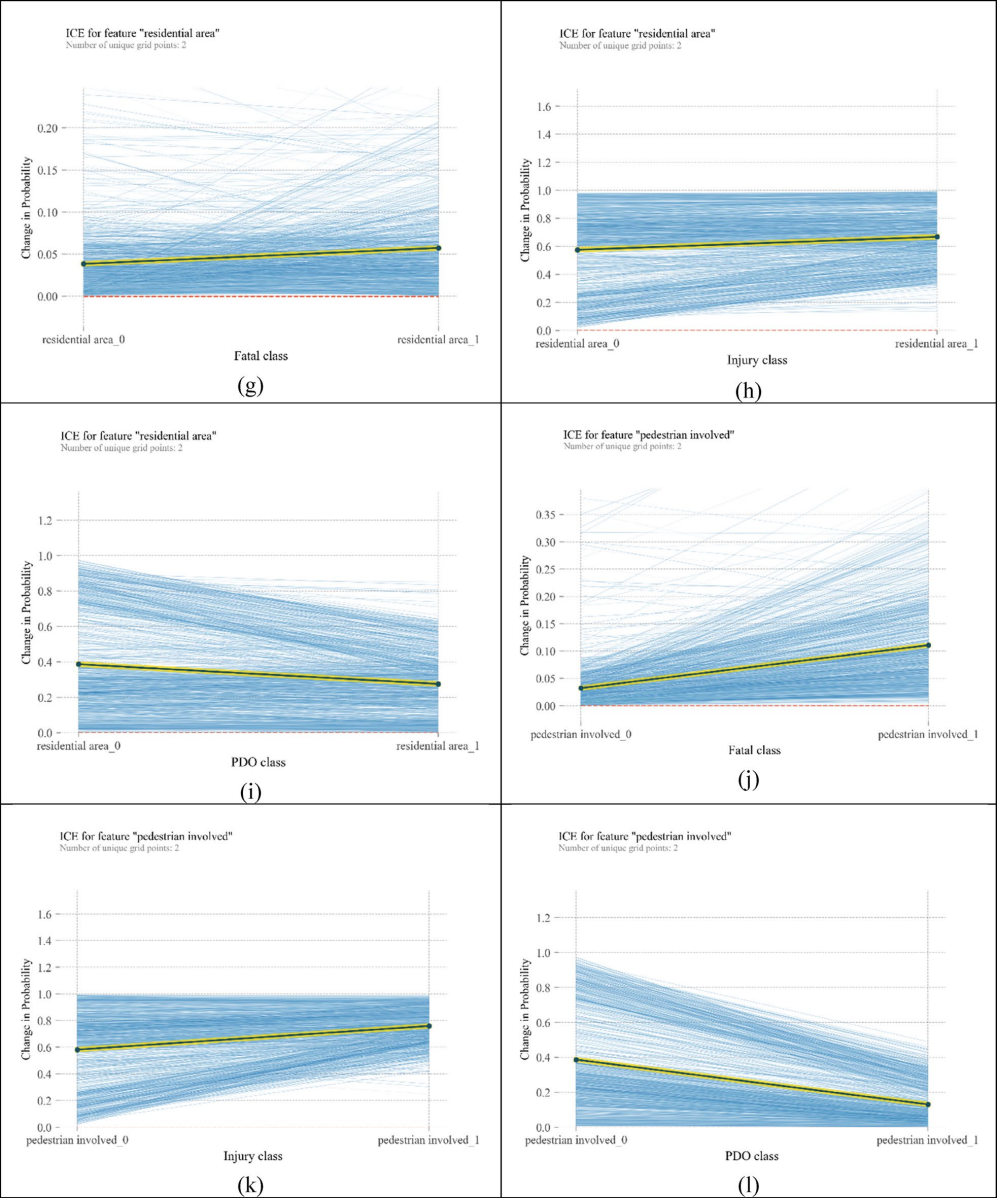
ICE plots for the most influencing variables on crash severity [plotted by Python Matplotlib v. 3.3.4 https://matplotlib.org].

ICE plots are developed for all observations in the train data set. Because of the large number of plots for each crash category (1141 curves), plots are depicted separately for each class in a single diagram. For the 4 most important variables, ICE plots are shown in Fig. 5. These plots show heterogeneity of crash severity. As observed in all plots in Fig. 5, all the curves start at various probabilities, and therefore the difference between observations is ambiguous. By cantering plots around a certain point, individual heterogeneity can be uncovered more reliably.

**Figure 6:**
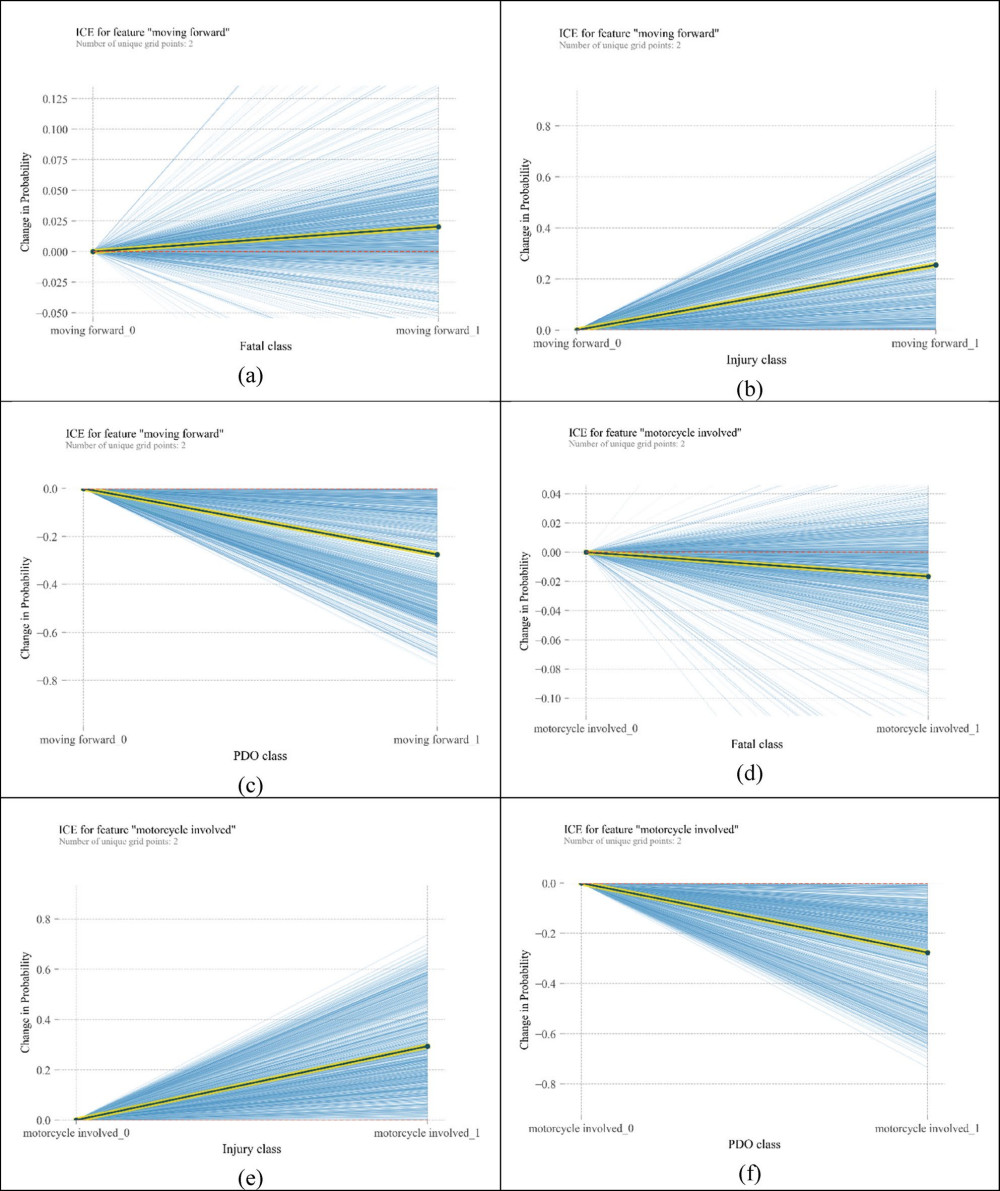

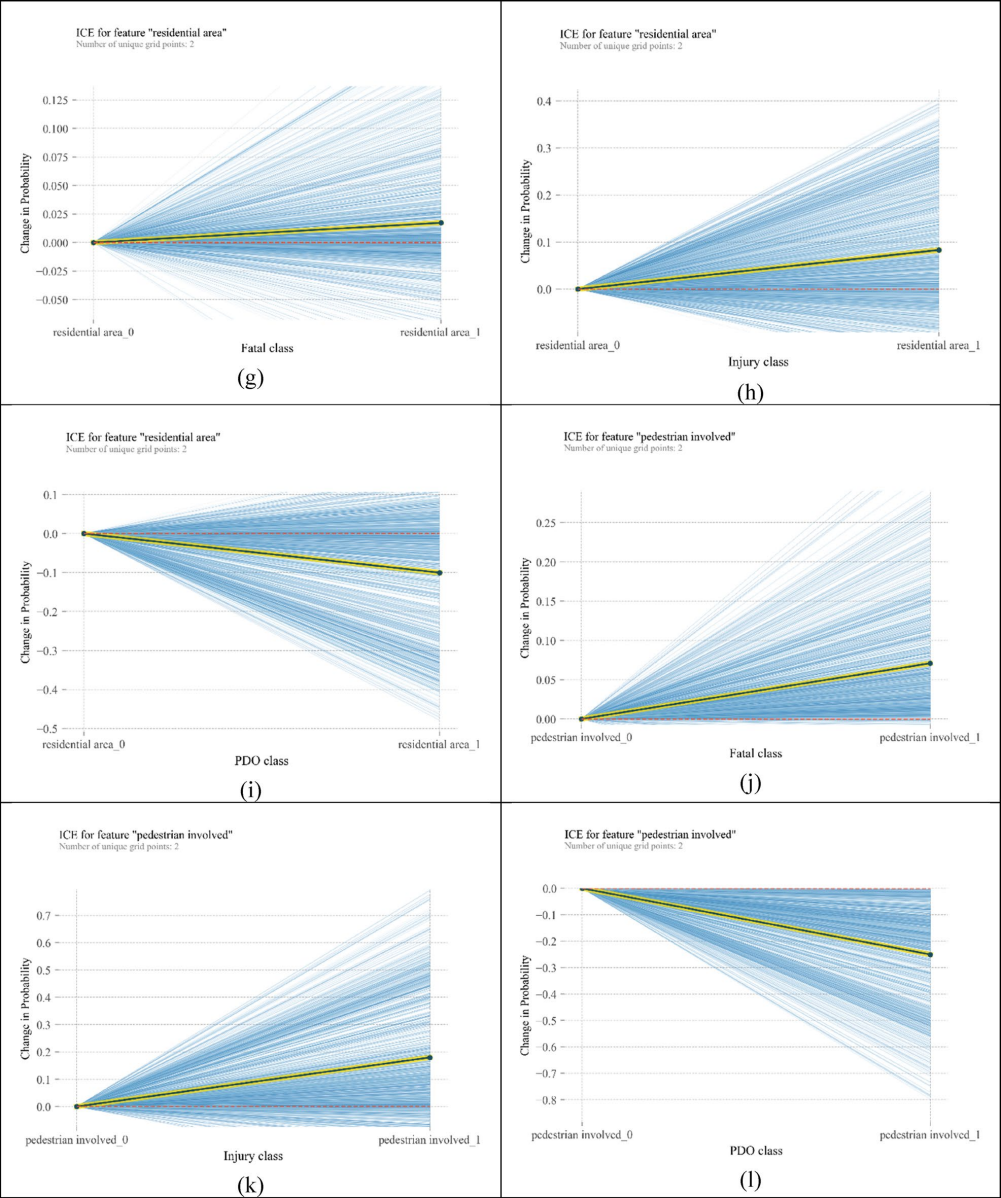
ICE plots for the most important variables [plotted by Python Matplotlib v. 3.3.4 https://matplotlib.org].

Centred ICE plots are shown in Fig. 6. Plots about “moving forward” show significant heterogeneity among observations, specifically in the fatal injury category. According to Fig. 6a, “moving forward” increases the probability of fatal crashes. From Fig. 6b, “moving forward” can double the probability of injury crashes; however, in some cases, this variable has no significant change in the probability of injury crashes. Figure 6c shows a heterogeneous but decreasing pattern of probability for the moving forward variable. It means that for all instances, getting involved in a crash while moving forward reduces its chance to be a PDO crash, but the amount of reduced probability differs. Similar heterogeneous behaviour can be seen for crashes involving monocycles in Fig. 6d–f. However, in Fig. 6d, the average trend is decreasing. “Residential area” repeats the behaviour of the two above mentioned variables, but the magnitudes of changes in probabilities are bigger. In addition, in injury and fatal classes, there are some curves whose slope is against the slope of PDP. In other words, “residential area” as the location of the crash reflects more heterogeneity than “moving forward” and “motorcycle” variables. In relation to “pedestrian involved” variable, when a pedestrian is hit by a vehicle in some instances, the chance of fatality crashes increases by 25%, and the chance of injury crashes increases by 70%. However, in a few observations, the probability of injury crashes decreases. In the PDO class, the probability of crash occurrence can decline by 70%.

### Interaction of PDPs

**Figure 7:**
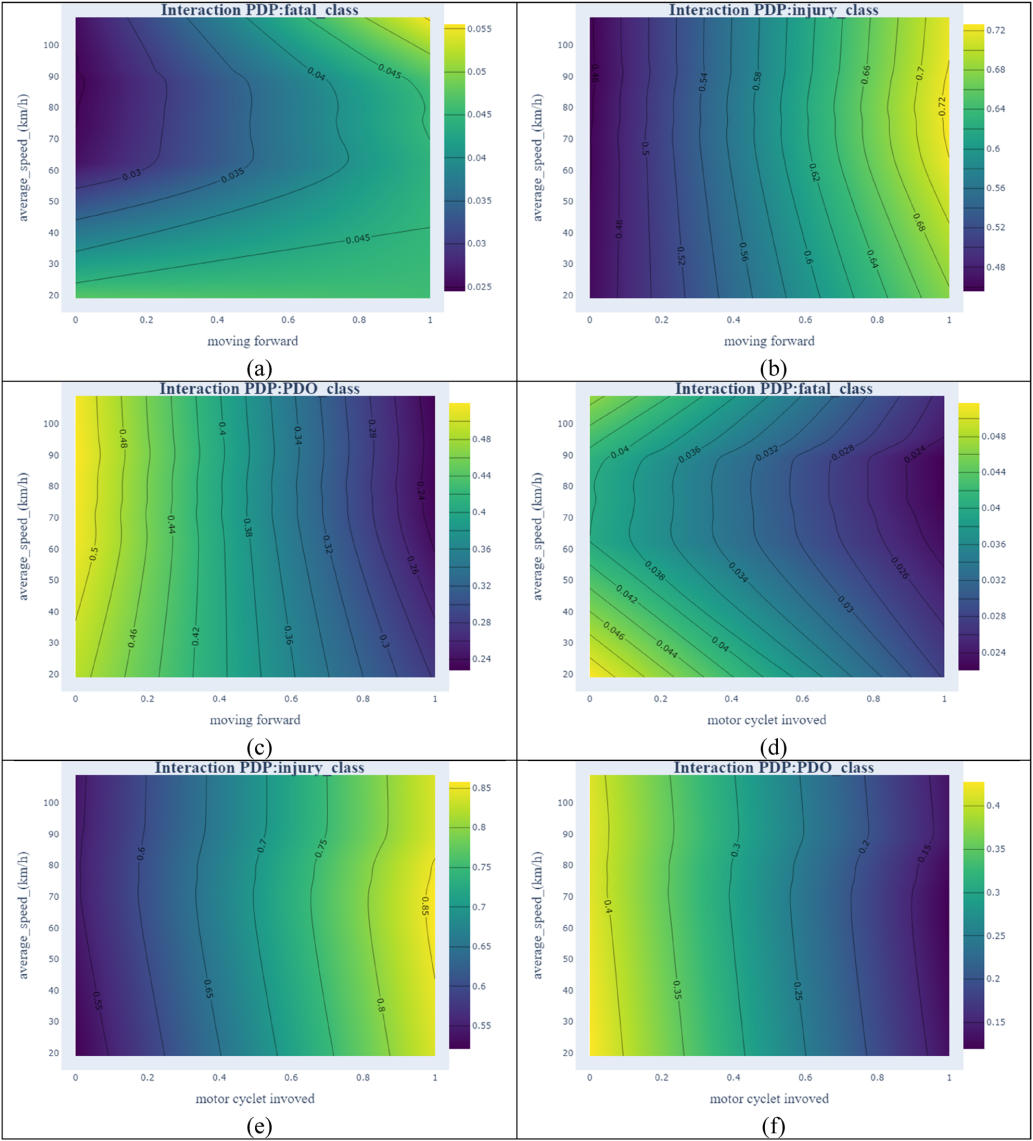

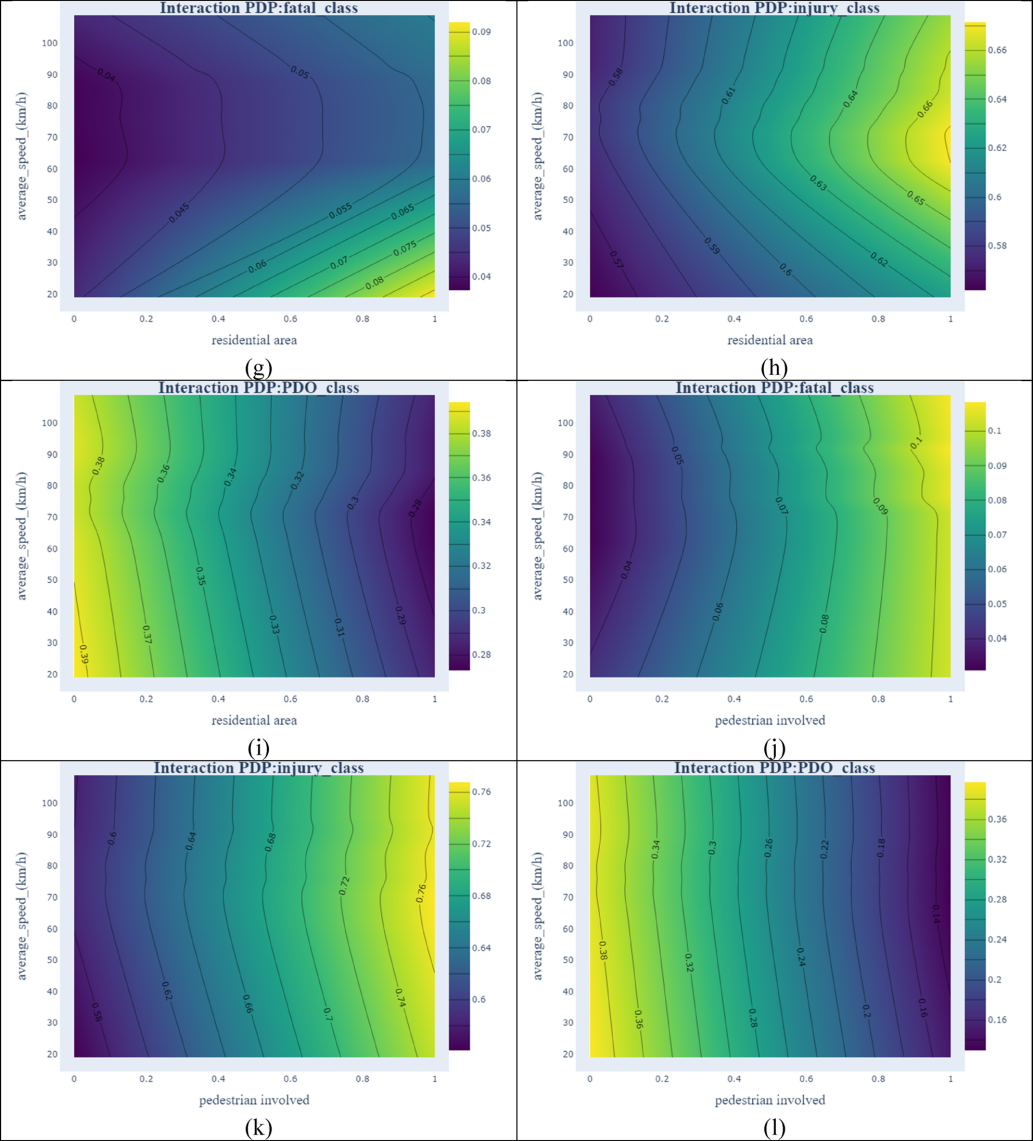
Interaction PDPs of selected variables with average speed [plotted by Plotly].

To uncover the interaction effect between independent variables, probability values by incorporating interaction effects are calculated. These values indicate how two variables could influence the dependent variable. Considering the interaction effects provide a better relationship between the dependent and independent variables^64^. Due to many possible plots for the combination of all variables for all categories of crashes ($$3*\left(\begin{array}{c}30\\ 2\end{array}\right)=1305$$), the interaction effects of the 4 most important variables and the average speed as the most important traffic-related variable are shown in Fig. 7.

In Fig. 7a, at lower speeds (from 20 to 40 km/h), by changing the moving direction to moving forward, there is no significant change in the probability of a fatal crash. However, at speeds of more than 90 km/h, the probability of fatal crashes is doubled. Almost parallel contours on the left side of Fig. 7b shows that changes in speed have a slight effect on the probability of injury crash, but its combination with moving forward could increase the probability of crash occurrence to 72% at speeds of 70 to 80 km/h. Smooth contours in Fig. 7c show that speed has no significant effect on the probability of a PDO crash. In Fig. 7d, the involvement of motorcycles in crashes decreases the probability of fatal crashes. This can be due to a few fatal crash observations that contain motorcycles (4 fatal crash observations in 215 traffic crashes involving motorcycles). In motorcycle crashes and at speeds between 60 and 70 km/h, the probability of injury crash reaches its maximum as shown in Fig. 7e; however, it decreases at lower speeds. From Fig. 7f, it can be observed that a combination of motorcycles and higher speeds slumps the probability of a PDO crash, despite the minor effect of speed when there are no motorcycles involved in a crash. When a crash occurs in residential sections of a rural area, the probability of having a fatal crash is reduced with the increase in the average speed (Fig. 7g). However, in Fig. 7h opposite situation is observed. High speed in residential areas can exacerbate the probability of injury crash to 0.66. In Fig. 7i, the probability of a PDO crash drops by the increase in speed and the existence of a residential area. However, vertical contours show that PDO crash probability is more dependent on the residential area than average speed. Figure 7j shows that fatal crash probability increases with pedestrian presence and speeding. At speeds around 70 km/h and when pedestrians are involved in the accident, the probability of injury crashes reaches the maximum value (Fig. 7k). The modest effect of speed on PDO crash probability can be derived from vertical contours in Fig. 7l.

Across severity categories, it is noticeable that fatal crashes are more influenced by “average speed” compared to other categories of crash severity. However, average speed does not have a substantial impact on PDO crashes which is shown in Fig. 7 by vertical and almost parallel contours. With respect to injury crashes, regardless of the second variable in all figures, at speeds of around 70 to 80 km/h, the probability of observing injury crashes reaches its maximum.

## Conclusions and future research directions

In this study, ERTs were trained to model crash severity using historical crash data in the Khorasan-Razavi province in Iran. A total of 1141 observations were used to train the model, and 285 instances were recruited to test the model. The train and test models achieved an accuracy of 85% and 81% and the F-measure values of 0.840 and 0.790, respectively. Feature importance analysis showed that the direction of movement alongside variables related to vulnerable users (e.g. pedestrians and motorcyclists) and land use variables (e.g. agricultural and residential areas) were found as the most influencing variables on crash severity. In addition, the traffic variables (e.g. average speed, traffic flow, headway) have more influence on crash severity compared to other variables.

PDPs were depicted to show the changes in values of variables that have an impact on the average probability of each crash category. To uncover the heterogeneity across dataset, individual exception plots were structured for each instance. Interaction of partial dependences was plotted to investigate the combined effect of variables on the probability of each crash category. These plots provide a useful measure to interpret the machine learning models. Using these plots would enable researchers to dissect the nonlinear relationship between dependent and independent variables.

The performance of the ERT model showed that this model could be an alternative to the existing well-known models in traffic safety such as random forest, Adaboost, gradient boosting, and XGBoost. The involvement of motorcycles was found to be the most influential factor in PDO and injury crashes. In fatal crashes, the involvement of pedestrians had the highest impact on increasing the probability of a fatal crash. Policy implications can be derived from interpretation results. However, this study is one of the first applications of the ERT in crash safety analysis in rural areas, and it should be applied to different datasets. On the other hand, in this study, four parameters of the model were tuned. However, the ERT contains other parameters that have the potential to be investigated in future studies.

## Data Availability

The sample dataset analysed during the current study are available in the GitHub repository at https://github.com/farshidafshar/ERTModel. The complete dataset is available from the corresponding author on reasonable request.

## Code availability

Code for data analysis is also available in the GitHub repository at https://github.com/farshidafshar/ERTModel.
